# Supplementary material for: Dual Functional S-Doped g-C3N4 Pinhole Porous Nanosheets for Selective Fluorescence Sensing of Ag+ and Visible-Light Photocatalysis of Dyes
Source: Molecules. 2019 Jan 27;24(3):450. doi: 10.3390/molecules24030450 (PMC6384794; doi:10.3390/molecules24030450)
Supplement: Supplementary file 1 [file molecules-24-00450-s001.pdf]

Supplementary data

for

Dual functional S-doped g-C_3N_4 pinhole porous nanosheets for selective fluorescence sensing of Ag^+ and visible-light photocatalysis of dyes

A. N. Kadam, Md. Moniruzzaman, Sang-Wha Lee*

Department of Chemical and Biological Engineering, Gachon University, 1342 Seongnamdaero, Seongnam-si, Republic of Korea

Correspondence: lswha@gachon.ac.kr (S.W. Lee)

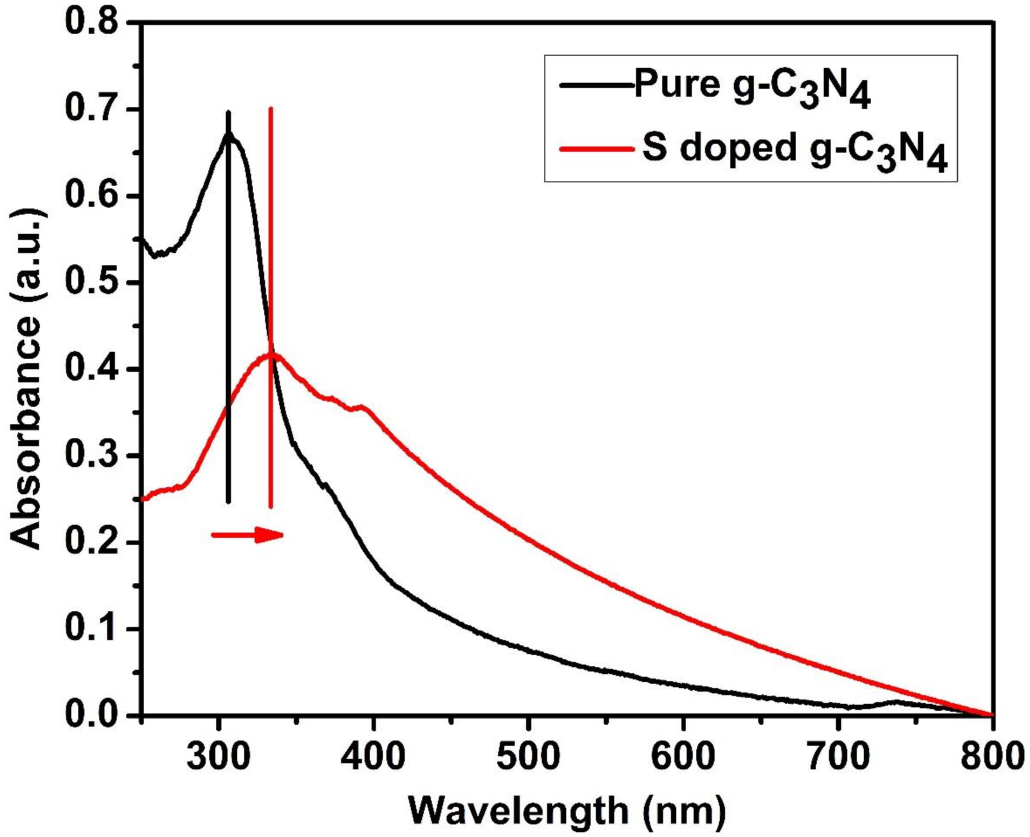

Figure S1. UV visible absorption spectra of pure g-C_3N_4 and S doped g-C_3N_4 in aqueous solution.

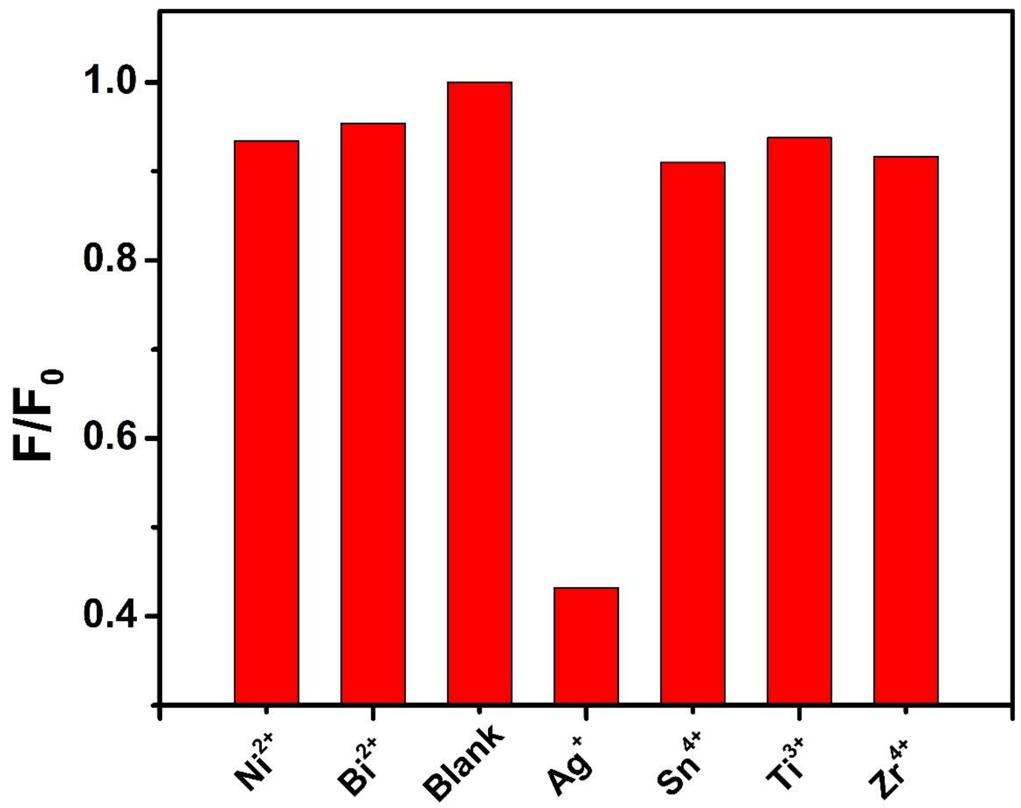

Figure S2. Selectivity of S doped g-C_3N_4 towards Ag^+ ions over various metal ions.

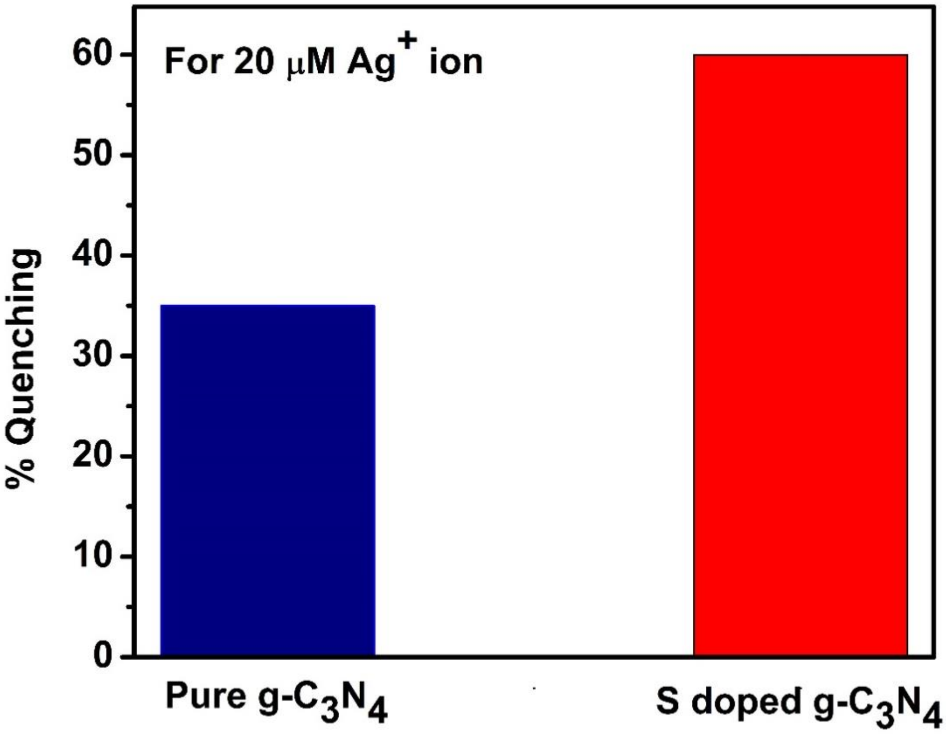

Figure S3. Fluorescence quenching% of pure g-C_3N_4 and S doped g-C_3N_4 solutions in the presence of 20 μM Ag^+ ions.

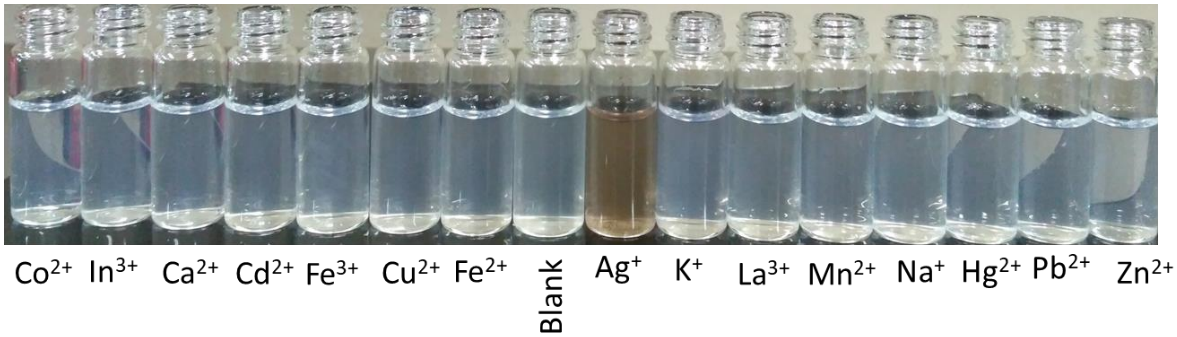

Figure S4. Pictorial image of all metal ions and Ag+ ion in g-C3N4 under UV light irradiation for 1 min.

(a)

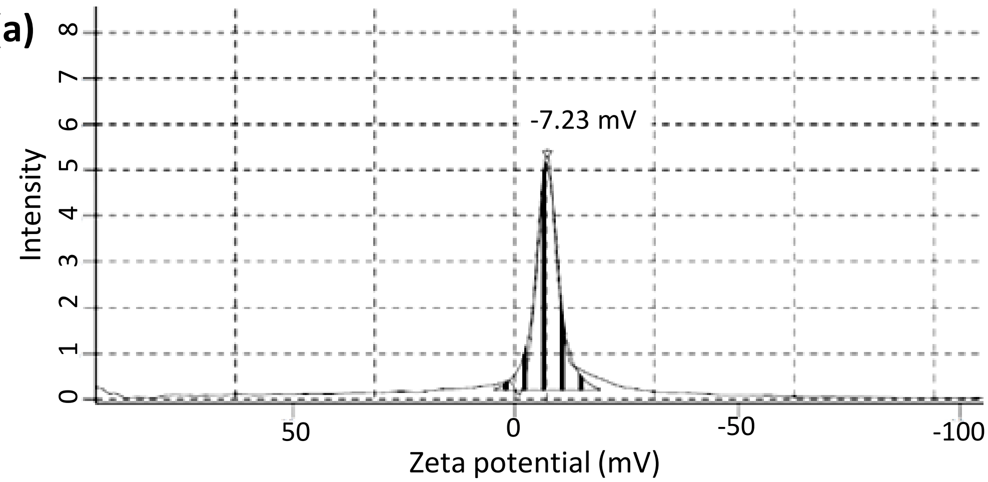

(b)

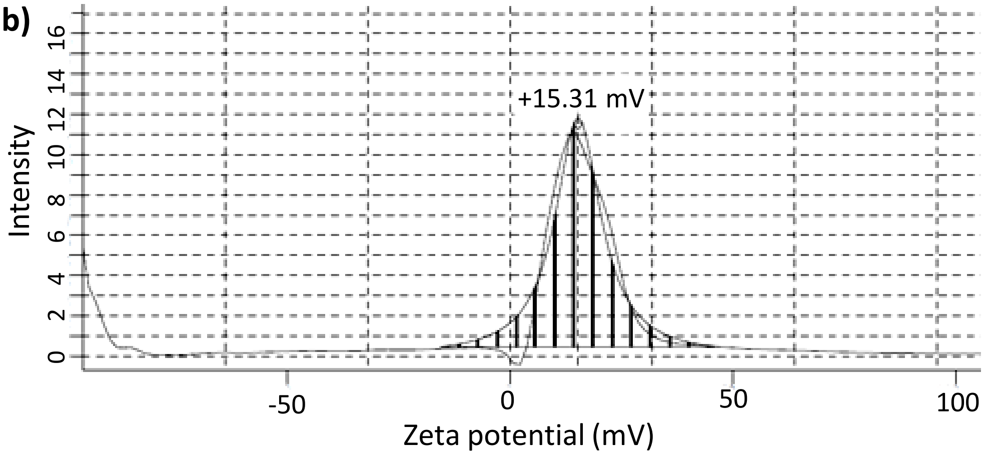

Figure S5. Zeta potentials of (a) pure SCNPNS, (b) SCNPNS-Ag^+ complexes. The concentration of Ag^+ in the mixed solution was 20 μM.

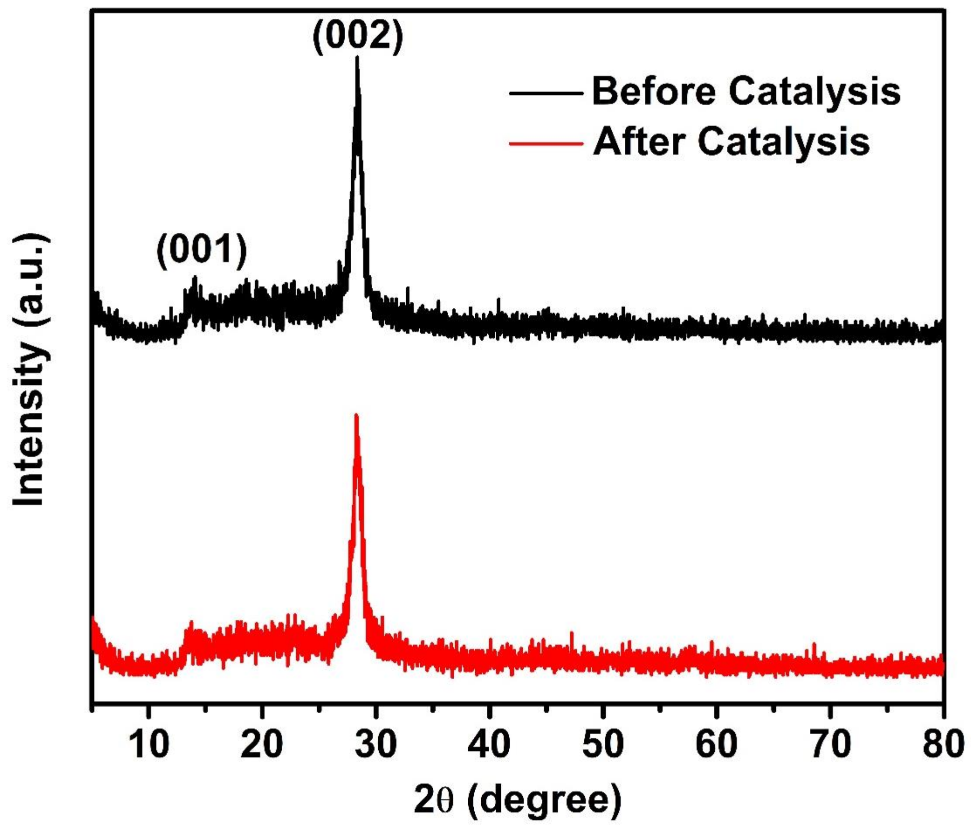

Figure S6. XRD patterns of the sample before and after the photocatalysis of SCNPNS towards MB under visible light.

Table S1. Double exponential fitting parameters of fluorescence life time decay curves of S doped g-C_3N_4 nanosheets before and after adding Ag^+ ions.

Compound	τ_1 / ns	B_1	τ_2 / ns	B_2	χ^2	τ_{ave} / ns
**S dopedg-C_3N_4**	2.1	0.642	5.2	0.358	1.05	3.89
**S dopedg-C_3N_4 + Ag^+**	1.9	0.518	4.68	0.482	0.95	3.83
